# 2-[(Adamantan-1-yl­amino)­meth­yl]phenol

**DOI:** 10.1107/S1600536811056194

**Published:** 2012-01-07

**Authors:** Ying-Chun Wang, Rong Tao

**Affiliations:** a College of Chemistry and Chemical Engineering, Southeast University, Nanjing 210096, People’s Republic of China

## Abstract

The asymmetric unit of the title compound, C_17_H_23_NO, contains two independent mol­ecules. In both mol­ecules, the hy­droxy group is involved in the formation of an intra­molecular O—H⋯N hydrogen bond. In the crystal, there are two crystallographically independent chains of the mol­ecules propagating along the *c* axis and formed by weak inter­molecular N—H⋯O hydrogen bonds.

## Related literature

For the ferroelectric properties of related amino derivatives, see: Fu *et al.* (2011*a* ▶,*b* ▶). For a related structure, see: Zhang *et al.* (2007 ▶).
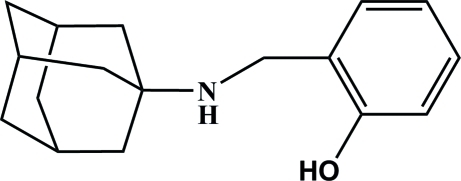


## Experimental

### 

#### Crystal data

C_17_H_23_NO
*M* *_r_* = 257.36
Monoclinic, 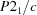

*a* = 23.451 (5) Å
*b* = 11.837 (2) Å
*c* = 10.684 (2) Å
β = 101.17 (3)°
*V* = 2909.6 (10) Å^3^
*Z* = 8
Mo *K*α radiation
μ = 0.07 mm^−1^
*T* = 298 K
0.10 × 0.05 × 0.05 mm

#### Data collection

Rigaku Mercury2 diffractometer
Absorption correction: multi-scan (*CrystalClear*; Rigaku, 2005 ▶) *T*_min_ = 0.910, *T*_max_ = 1.000
27928 measured reflections
6616 independent reflections
3011 reflections with *I* > 2σ(*I*)
*R*_int_ = 0.105

#### Refinement

*R*[*F* ^2^ > 2σ(*F* ^2^)] = 0.074
*wR*(*F* ^2^) = 0.178
*S* = 1.02
6616 reflections
344 parameters
6 restraints
H-atom parameters constrained
Δρ_max_ = 0.35 e Å^−3^
Δρ_min_ = −0.17 e Å^−3^

### 

Data collection: *CrystalClear* (Rigaku, 2005 ▶); cell refinement: *CrystalClear*; data reduction: *CrystalClear*; program(s) used to solve structure: *SHELXS97* (Sheldrick, 2008 ▶); program(s) used to refine structure: *SHELXL97* (Sheldrick, 2008 ▶); molecular graphics: *SHELXTL* (Sheldrick, 2008 ▶); software used to prepare material for publication: *SHELXTL*.

## Supplementary Material

Crystal structure: contains datablock(s) I, global. DOI: 10.1107/S1600536811056194/cv5227sup1.cif

Structure factors: contains datablock(s) I. DOI: 10.1107/S1600536811056194/cv5227Isup2.hkl

Supplementary material file. DOI: 10.1107/S1600536811056194/cv5227Isup3.cml

Additional supplementary materials:  crystallographic information; 3D view; checkCIF report

## Figures and Tables

**Table 1:** Hydrogen-bond geometry (Å, °)

*D*—H⋯*A*	*D*—H	H⋯*A*	*D*⋯*A*	*D*—H⋯*A*
O2—H2*B*⋯N2	0.82	1.95	2.690 (3)	151
O1—H1*B*⋯N1	0.82	1.92	2.670 (3)	152
N2—H2*A*⋯O2^i^	0.89	2.64	3.496 (3)	161
N1—H1*A*⋯O1^i^	0.89	2.50	3.344 (3)	158

Symmetry code: (i) 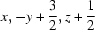.

**Fig. 1.**
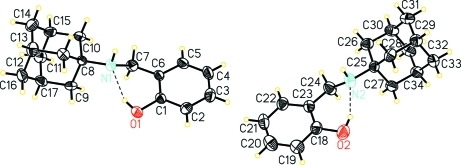
A content of the asymmetric unit of (I) with the atomic numbering scheme. Displacement ellipsoids are drawn at the 30% probability level. Dashed lines denote intramolecular hydrogen bonds.

## supplementary crystallographic information

### Comment

Simple organic salts containig amino cations have attracted an attention as materials which display ferroelectric-paraelectric phase transitions (Fu *et al.*, 2011*a*,*b*, and references therein). Herewith we present the crystal structure of the title compound (I), which can be used as cation in organic salts.

The asymmetric part of (I) contains two independent molecules (Fig. 1). All bond lengths and angles are normal and comparable with those reported for the cation in N-(2-pyridylmethyl)adamantane-1-ammonium chloride monohydrate (Zhang *et al.*, 2007). The hydroxyl O (O1 and O2) atoms are involved in hydrogen bonds (Table 1) with the amino N atoms (N1 and N2) with the O—H···N distance of 2.670 (3) and 2.694 (3) Å, respectively. These H-bond interactions build a R^2^_1_(6) ring which play an important role in stabilizing the structural conformation (Table 1).

In the crystal structure, there are two crystallographically independent chains of the molecules propagated along *c* axis and formed by the weak intermolecular N—H···O hydrogen bonds (Table 1).

### Experimental

Salicylaldehyde (2.44 g, 20 mmol) and KOH (1.12 g, 20 mmol) were added into a solution of amantadine hydrochloride (3.76 g, 20 mmol) in ethanol. Then a little of anhydrous magnesium sulfate was added into it, after 6 h return the yellow precipitate came out. The yellow solid of amantadine shrink Yang Schiff was obtained by filtration, collection and drying. NaBH_4_ (3.78 g, 10 mmol) was added into a solution of amantadine shrink Yang Schiff (6.38 g, 25 mmol) in anhydrous methanol (120 ml). After 5 h reaction, then the white solid, N-(2-Hydroxybenzyl)adamantan-1-amine was obtained by reduced pressure distillation, extraction and drying. The N-(2-Hydroxybenzyl)adamantan-1-amine (3 mmol) was dissolved in water/EtOH (1:1 *v*/*v*) solution. The solvent was slowly evaporated in air affording colourless block-shaped crystals of the title compound suitable for X-ray analysis.

### Refinement

C-bound H atoms were fixed geometrically [C—H 0.93–0.98 Å], and treated as riding, with *U*_iso_(H) = 1.2*U*_eq_(C). H atoms bonded to N and O atoms were located in difference Fourier maps and restrained to H—N = 0.89 (2) Å and H—O = 0.82 (2) Å. In the last stage of the refinement, they were treated as riding, with *U*_iso_(H) = 1.2*U*_eq_(N, O).

### Crystal data

**Table:**

C_17_H_23_NO	*F*(000) = 1120
*M**_r_* = 257.36	*D*_x_ = 1.175 Mg m^−^^3^
Monoclinic, *P*2_1_/*c*	Mo *K*α radiation, λ = 0.71073 Å
Hall symbol: -P 2ybc	Cell parameters from 6616 reflections
*a* = 23.451 (5) Å	θ = 3.2–27.5°
*b* = 11.837 (2) Å	µ = 0.07 mm^−^^1^
*c* = 10.684 (2) Å	*T* = 298 K
β = 101.17 (3)°	Block, colourless
*V* = 2909.6 (10) Å^3^	0.10 × 0.05 × 0.05 mm
*Z* = 8	

### Data collection

**Table:**

Rigaku Mercury2 diffractometer	6616 independent reflections
Radiation source: fine-focus sealed tube	3011 reflections with *I* > 2σ(*I*)
graphite	*R*_int_ = 0.105
Detector resolution: 13.6612 pixels mm^-1^	θ_max_ = 27.5°, θ_min_ = 3.2°
CCD profile fitting scans	*h* = −30→30
Absorption correction: multi-scan (*CrystalClear*; Rigaku, 2005)	*k* = −15→14
*T*_min_ = 0.910, *T*_max_ = 1.000	*l* = −13→13
27928 measured reflections	

### Refinement

**Table:**

Refinement on *F*^2^	Secondary atom site location: difference Fourier map
Least-squares matrix: full	Hydrogen site location: inferred from neighbouring sites
*R*[*F*^2^ > 2σ(*F*^2^)] = 0.074	H-atom parameters constrained
*wR*(*F*^2^) = 0.178	*w* = 1/[σ^2^(*F*_o_^2^) + (0.0533*P*)^2^ + 0.4843*P*] where *P* = (*F*_o_^2^ + 2*F*_c_^2^)/3
*S* = 1.02	(Δ/σ)_max_ < 0.001
6616 reflections	Δρ_max_ = 0.35 e Å^−^^3^
344 parameters	Δρ_min_ = −0.17 e Å^−^^3^
6 restraints	Extinction correction: *SHELXL97* (Sheldrick, 2008), Fc^*^=kFc[1+0.001xFc^2^λ^3^/sin(2θ)]^-1/4^
Primary atom site location: structure-invariant direct methods	Extinction coefficient: 0.0053 (6)

### Special details

**Table:**

Geometry. All esds (except the esd in the dihedral angle between two l.s. planes) are estimated using the full covariance matrix. The cell esds are taken into account individually in the estimation of esds in distances, angles and torsion angles; correlations between esds in cell parameters are only used when they are defined by crystal symmetry. An approximate (isotropic) treatment of cell esds is used for estimating esds involving l.s. planes.
Refinement. Refinement of F^2^ against ALL reflections. The weighted R-factor wR and goodness of fit S are based on F^2^, conventional R-factors R are based on F, with F set to zero for negative F^2^. The threshold expression of F^2^ > 2sigma(F^2^) is used only for calculating R-factors(gt) etc. and is not relevant to the choice of reflections for refinement. R-factors based on F^2^ are statistically about twice as large as those based on F, and R- factors based on ALL data will be even larger.

### Fractional atomic coordinates and isotropic or equivalent isotropic displacement parameters (Å^2^)

**Table:**

	*x*	*y*	*z*	*U*_iso_*/*U*_eq_	
O2	0.52444 (9)	0.72475 (18)	0.0364 (2)	0.0865 (7)	
H2B	0.5043	0.7196	0.0909	0.104*	
N2	0.48436 (9)	0.64480 (17)	0.2387 (2)	0.0697 (7)	
H2A	0.5005	0.6872	0.3050	0.084*	
C22	0.63107 (13)	0.5440 (3)	0.2346 (3)	0.0679 (9)	
H22A	0.6337	0.4904	0.2991	0.081*	
C21	0.68075 (13)	0.5737 (3)	0.1911 (4)	0.0796 (10)	
H21A	0.7161	0.5396	0.2251	0.095*	
C20	0.67765 (16)	0.6536 (3)	0.0976 (4)	0.0829 (10)	
H20A	0.7111	0.6743	0.0688	0.100*	
C19	0.62562 (15)	0.7033 (3)	0.0459 (3)	0.0726 (9)	
H19A	0.6237	0.7568	−0.0185	0.087*	
C18	0.57589 (13)	0.6735 (3)	0.0903 (3)	0.0630 (8)	
C23	0.57746 (12)	0.5917 (2)	0.1850 (3)	0.0578 (8)	
C24	0.52176 (13)	0.5513 (2)	0.2236 (3)	0.0772 (10)	
H24A	0.5014	0.5003	0.1589	0.093*	
H24B	0.5313	0.5099	0.3033	0.093*	
C26	0.42163 (13)	0.5279 (3)	0.3580 (3)	0.0682 (9)	
H26A	0.4369	0.4571	0.3326	0.082*	
H26B	0.4455	0.5511	0.4384	0.082*	
C25	0.42430 (9)	0.6185 (2)	0.2558 (2)	0.0529 (7)	
C30	0.35931 (14)	0.5110 (3)	0.3751 (3)	0.0701 (9)	
H30A	0.3583	0.4535	0.4406	0.084*	
C31	0.33580 (14)	0.6222 (3)	0.4164 (3)	0.0763 (9)	
H31A	0.3592	0.6464	0.4969	0.092*	
H31B	0.2962	0.6117	0.4284	0.092*	
C29	0.32235 (14)	0.4721 (3)	0.2490 (3)	0.0803 (10)	
H29A	0.2826	0.4595	0.2594	0.096*	
H29B	0.3375	0.4015	0.2226	0.096*	
C32	0.33722 (12)	0.7125 (3)	0.3147 (3)	0.0636 (8)	
H32A	0.3222	0.7840	0.3415	0.076*	
C33	0.30014 (12)	0.6745 (3)	0.1893 (3)	0.0738 (9)	
H33A	0.3007	0.7318	0.1247	0.089*	
H33B	0.2603	0.6644	0.1996	0.089*	
C34	0.32371 (12)	0.5633 (3)	0.1473 (3)	0.0710 (9)	
H34A	0.2997	0.5391	0.0663	0.085*	
C27	0.38680 (12)	0.5796 (3)	0.1301 (3)	0.0628 (8)	
H27A	0.4018	0.5091	0.1034	0.075*	
H27B	0.3881	0.6356	0.0644	0.075*	
C28	0.39965 (12)	0.7287 (2)	0.2963 (3)	0.0608 (8)	
H28A	0.4006	0.7859	0.2317	0.073*	
H28B	0.4235	0.7548	0.3755	0.073*	
O1	0.94801 (7)	0.73205 (15)	0.46936 (17)	0.0605 (5)	
H1B	0.9695	0.7223	0.5388	0.073*	
N1	0.98628 (8)	0.66076 (17)	0.70798 (19)	0.0477 (6)	
H1A	0.9719	0.7045	0.7622	0.057*	
C8	1.04523 (10)	0.6247 (2)	0.7736 (2)	0.0394 (6)	
C11	1.07657 (11)	0.7317 (2)	0.8317 (3)	0.0519 (7)	
H11A	1.0781	0.7862	0.7647	0.062*	
H11B	1.0551	0.7652	0.8912	0.062*	
C10	1.04375 (11)	0.5392 (2)	0.8817 (2)	0.0502 (7)	
H10A	1.0220	0.5708	0.9420	0.060*	
H10B	1.0243	0.4707	0.8465	0.060*	
C15	1.10557 (12)	0.5113 (2)	0.9503 (2)	0.0563 (7)	
H15A	1.1040	0.4569	1.0187	0.068*	
C14	1.13544 (13)	0.6183 (3)	1.0073 (3)	0.0716 (9)	
H14A	1.1745	0.6007	1.0520	0.086*	
H14B	1.1142	0.6503	1.0681	0.086*	
C9	1.07946 (11)	0.5733 (2)	0.6797 (2)	0.0493 (7)	
H9A	1.0810	0.6266	0.6114	0.059*	
H9B	1.0601	0.5054	0.6424	0.059*	
C17	1.14125 (12)	0.5444 (2)	0.7484 (3)	0.0572 (8)	
H17A	1.1629	0.5117	0.6874	0.069*	
C16	1.13907 (13)	0.4596 (2)	0.8554 (3)	0.0648 (8)	
H16A	1.1201	0.3907	0.8200	0.078*	
H16B	1.1782	0.4407	0.8985	0.078*	
C13	1.13800 (12)	0.7038 (2)	0.9008 (3)	0.0644 (8)	
H13A	1.1575	0.7728	0.9374	0.077*	
C12	1.17164 (12)	0.6521 (3)	0.8053 (3)	0.0710 (9)	
H12A	1.1736	0.7059	0.7378	0.085*	
H12B	1.2110	0.6346	0.8481	0.085*	
C7	0.94311 (11)	0.5709 (2)	0.6667 (3)	0.0555 (7)	
H7A	0.9324	0.5360	0.7410	0.067*	
H7B	0.9601	0.5132	0.6208	0.067*	
C6	0.88973 (10)	0.6180 (2)	0.5821 (2)	0.0444 (6)	
C1	0.89467 (11)	0.6952 (2)	0.4859 (2)	0.0460 (6)	
C2	0.84582 (12)	0.7356 (2)	0.4048 (3)	0.0548 (7)	
H2C	0.8497	0.7870	0.3411	0.066*	
C3	0.79137 (12)	0.7001 (3)	0.4181 (3)	0.0644 (8)	
H3A	0.7585	0.7277	0.3637	0.077*	
C4	0.78559 (12)	0.6236 (3)	0.5118 (3)	0.0680 (9)	
H4A	0.7489	0.5989	0.5204	0.082*	
C5	0.83443 (12)	0.5836 (2)	0.5929 (3)	0.0590 (8)	
H5A	0.8301	0.5324	0.6564	0.071*	

### Atomic displacement parameters (Å^2^)

**Table:**

	*U*^11^	*U*^22^	*U*^33^	*U*^12^	*U*^13^	*U*^23^
O2	0.0738 (15)	0.0910 (17)	0.0903 (17)	0.0089 (13)	0.0055 (12)	0.0293 (13)
N2	0.0572 (15)	0.0592 (16)	0.093 (2)	0.0038 (13)	0.0145 (13)	0.0019 (14)
C22	0.063 (2)	0.071 (2)	0.067 (2)	0.0103 (17)	0.0067 (16)	−0.0087 (16)
C21	0.049 (2)	0.087 (3)	0.100 (3)	0.0019 (19)	0.0056 (18)	−0.029 (2)
C20	0.071 (3)	0.073 (2)	0.110 (3)	−0.022 (2)	0.029 (2)	−0.024 (2)
C19	0.077 (2)	0.059 (2)	0.084 (2)	−0.0111 (19)	0.0202 (19)	−0.0016 (17)
C18	0.0575 (19)	0.062 (2)	0.067 (2)	−0.0019 (17)	0.0049 (16)	−0.0052 (16)
C23	0.0530 (18)	0.0577 (19)	0.062 (2)	0.0031 (15)	0.0087 (14)	−0.0030 (15)
C24	0.076 (2)	0.070 (2)	0.092 (2)	0.0194 (19)	0.0320 (18)	0.0160 (18)
C26	0.080 (2)	0.062 (2)	0.063 (2)	0.0060 (17)	0.0135 (16)	0.0113 (15)
C25	0.0434 (16)	0.0581 (19)	0.0554 (18)	−0.0024 (14)	0.0051 (13)	0.0051 (14)
C30	0.087 (2)	0.064 (2)	0.066 (2)	−0.0076 (18)	0.0287 (18)	0.0088 (17)
C31	0.090 (2)	0.085 (2)	0.059 (2)	−0.008 (2)	0.0253 (17)	−0.0076 (18)
C29	0.082 (2)	0.076 (2)	0.092 (3)	−0.0284 (19)	0.039 (2)	−0.026 (2)
C32	0.065 (2)	0.063 (2)	0.063 (2)	0.0021 (16)	0.0123 (15)	−0.0124 (16)
C33	0.0521 (18)	0.099 (3)	0.071 (2)	0.0024 (18)	0.0116 (16)	−0.0004 (19)
C34	0.0564 (19)	0.103 (3)	0.0528 (19)	−0.0197 (18)	0.0077 (14)	−0.0229 (18)
C27	0.0643 (19)	0.072 (2)	0.0562 (19)	−0.0018 (16)	0.0203 (14)	−0.0035 (15)
C28	0.0628 (19)	0.0510 (18)	0.0630 (19)	−0.0011 (15)	−0.0021 (14)	−0.0030 (14)
O1	0.0493 (11)	0.0734 (14)	0.0611 (12)	−0.0100 (10)	0.0166 (9)	0.0133 (10)
N1	0.0467 (13)	0.0445 (13)	0.0521 (14)	−0.0005 (11)	0.0101 (10)	−0.0030 (10)
C8	0.0375 (14)	0.0432 (15)	0.0391 (15)	0.0019 (12)	0.0114 (11)	0.0031 (11)
C11	0.0559 (17)	0.0402 (16)	0.0623 (18)	−0.0004 (14)	0.0181 (14)	−0.0035 (13)
C10	0.0551 (17)	0.0546 (17)	0.0454 (16)	0.0022 (14)	0.0208 (13)	0.0047 (13)
C15	0.0672 (19)	0.0598 (19)	0.0434 (17)	0.0112 (16)	0.0145 (14)	0.0122 (14)
C14	0.070 (2)	0.085 (2)	0.055 (2)	0.0081 (18)	0.0008 (15)	−0.0055 (17)
C9	0.0637 (18)	0.0503 (17)	0.0370 (15)	0.0046 (14)	0.0175 (13)	0.0009 (12)
C17	0.0535 (17)	0.065 (2)	0.0597 (19)	0.0126 (15)	0.0276 (14)	0.0036 (15)
C16	0.070 (2)	0.0611 (19)	0.065 (2)	0.0194 (16)	0.0172 (15)	0.0106 (15)
C13	0.0584 (19)	0.0519 (19)	0.079 (2)	−0.0080 (15)	0.0037 (16)	−0.0125 (16)
C12	0.0430 (16)	0.084 (2)	0.087 (2)	0.0001 (17)	0.0150 (16)	0.0176 (19)
C7	0.0597 (18)	0.0498 (17)	0.0577 (18)	−0.0136 (15)	0.0132 (14)	0.0037 (14)
C6	0.0381 (14)	0.0512 (17)	0.0456 (16)	−0.0066 (13)	0.0128 (12)	−0.0078 (13)
C1	0.0412 (15)	0.0479 (16)	0.0505 (17)	−0.0055 (13)	0.0133 (12)	−0.0060 (13)
C2	0.0595 (19)	0.0509 (17)	0.0520 (18)	−0.0026 (15)	0.0062 (14)	−0.0047 (13)
C3	0.0495 (19)	0.070 (2)	0.070 (2)	0.0025 (16)	0.0018 (15)	−0.0165 (17)
C4	0.0418 (17)	0.084 (2)	0.080 (2)	−0.0109 (17)	0.0167 (16)	−0.0117 (19)
C5	0.0534 (18)	0.070 (2)	0.0577 (19)	−0.0108 (16)	0.0208 (14)	−0.0018 (15)

### Geometric parameters (Å, °)

**Table:**

O2—C18	1.373 (3)	O1—C1	1.368 (3)
O2—H2B	0.8200	O1—H1B	0.8200
N2—C24	1.441 (2)	N1—C7	1.475 (3)
N2—C25	1.488 (2)	N1—C8	1.486 (3)
N2—H2A	0.8900	N1—H1A	0.8901
C22—C21	1.381 (4)	C8—C9	1.527 (3)
C22—C23	1.387 (4)	C8—C11	1.534 (3)
C22—H22A	0.9300	C8—C10	1.541 (3)
C21—C20	1.366 (5)	C11—C13	1.522 (4)
C21—H21A	0.9300	C11—H11A	0.9700
C20—C19	1.371 (4)	C11—H11B	0.9700
C20—H20A	0.9300	C10—C15	1.529 (3)
C19—C18	1.387 (4)	C10—H10A	0.9700
C19—H19A	0.9300	C10—H10B	0.9700
C18—C23	1.396 (4)	C15—C14	1.516 (4)
C23—C24	1.521 (4)	C15—C16	1.526 (4)
C24—H24A	0.9700	C15—H15A	0.9800
C24—H24B	0.9700	C14—C13	1.533 (4)
C26—C30	1.521 (4)	C14—H14A	0.9700
C26—C25	1.541 (4)	C14—H14B	0.9700
C26—H26A	0.9700	C9—C17	1.531 (3)
C26—H26B	0.9700	C9—H9A	0.9700
C25—C28	1.524 (4)	C9—H9B	0.9700
C25—C27	1.527 (4)	C17—C12	1.528 (4)
C30—C29	1.524 (4)	C17—C16	1.530 (4)
C30—C31	1.525 (4)	C17—H17A	0.9800
C30—H30A	0.9800	C16—H16A	0.9700
C31—C32	1.529 (4)	C16—H16B	0.9700
C31—H31A	0.9700	C13—C12	1.533 (4)
C31—H31B	0.9700	C13—H13A	0.9800
C29—C34	1.536 (4)	C12—H12A	0.9700
C29—H29A	0.9700	C12—H12B	0.9700
C29—H29B	0.9700	C7—C6	1.502 (3)
C32—C33	1.517 (4)	C7—H7A	0.9700
C32—C28	1.527 (4)	C7—H7B	0.9700
C32—H32A	0.9800	C6—C5	1.385 (3)
C33—C34	1.529 (4)	C6—C1	1.396 (3)
C33—H33A	0.9700	C1—C2	1.382 (3)
C33—H33B	0.9700	C2—C3	1.378 (4)
C34—C27	1.538 (4)	C2—H2C	0.9300
C34—H34A	0.9800	C3—C4	1.376 (4)
C27—H27A	0.9700	C3—H3A	0.9300
C27—H27B	0.9700	C4—C5	1.379 (4)
C28—H28A	0.9700	C4—H4A	0.9300
C28—H28B	0.9700	C5—H5A	0.9300
			
C18—O2—H2B	104.5	C1—O1—H1B	104.8
C24—N2—C25	117.7 (2)	C7—N1—C8	117.1 (2)
C24—N2—H2A	110.4	C7—N1—H1A	106.8
C25—N2—H2A	106.7	C8—N1—H1A	107.2
C21—C22—C23	121.9 (3)	N1—C8—C9	111.34 (19)
C21—C22—H22A	119.0	N1—C8—C11	106.42 (19)
C23—C22—H22A	119.0	C9—C8—C11	108.84 (19)
C20—C21—C22	119.6 (3)	N1—C8—C10	112.84 (19)
C20—C21—H21A	120.2	C9—C8—C10	108.8 (2)
C22—C21—H21A	120.2	C11—C8—C10	108.5 (2)
C21—C20—C19	120.6 (3)	C13—C11—C8	110.6 (2)
C21—C20—H20A	119.7	C13—C11—H11A	109.5
C19—C20—H20A	119.7	C8—C11—H11A	109.5
C20—C19—C18	119.7 (3)	C13—C11—H11B	109.5
C20—C19—H19A	120.2	C8—C11—H11B	109.5
C18—C19—H19A	120.2	H11A—C11—H11B	108.1
O2—C18—C19	118.3 (3)	C15—C10—C8	110.2 (2)
O2—C18—C23	120.5 (3)	C15—C10—H10A	109.6
C19—C18—C23	121.2 (3)	C8—C10—H10A	109.6
C22—C23—C18	117.0 (3)	C15—C10—H10B	109.6
C22—C23—C24	122.0 (3)	C8—C10—H10B	109.6
C18—C23—C24	120.8 (3)	H10A—C10—H10B	108.1
N2—C24—C23	111.2 (2)	C14—C15—C16	110.1 (2)
N2—C24—H24A	109.4	C14—C15—C10	109.5 (2)
C23—C24—H24A	109.4	C16—C15—C10	109.3 (2)
N2—C24—H24B	109.4	C14—C15—H15A	109.3
C23—C24—H24B	109.4	C16—C15—H15A	109.3
H24A—C24—H24B	108.0	C10—C15—H15A	109.3
C30—C26—C25	110.4 (2)	C15—C14—C13	109.6 (2)
C30—C26—H26A	109.6	C15—C14—H14A	109.8
C25—C26—H26A	109.6	C13—C14—H14A	109.8
C30—C26—H26B	109.6	C15—C14—H14B	109.8
C25—C26—H26B	109.6	C13—C14—H14B	109.8
H26A—C26—H26B	108.1	H14A—C14—H14B	108.2
N2—C25—C28	106.01 (19)	C8—C9—C17	110.1 (2)
N2—C25—C27	110.7 (2)	C8—C9—H9A	109.6
C28—C25—C27	108.8 (2)	C17—C9—H9A	109.6
N2—C25—C26	113.7 (2)	C8—C9—H9B	109.6
C28—C25—C26	109.0 (2)	C17—C9—H9B	109.6
C27—C25—C26	108.6 (2)	H9A—C9—H9B	108.2
C26—C30—C29	109.2 (2)	C12—C17—C16	109.3 (2)
C26—C30—C31	109.3 (3)	C12—C17—C9	109.3 (2)
C29—C30—C31	109.9 (3)	C16—C17—C9	109.8 (2)
C26—C30—H30A	109.5	C12—C17—H17A	109.5
C29—C30—H30A	109.5	C16—C17—H17A	109.5
C31—C30—H30A	109.5	C9—C17—H17A	109.5
C30—C31—C32	109.8 (2)	C15—C16—C17	109.2 (2)
C30—C31—H31A	109.7	C15—C16—H16A	109.8
C32—C31—H31A	109.7	C17—C16—H16A	109.8
C30—C31—H31B	109.7	C15—C16—H16B	109.8
C32—C31—H31B	109.7	C17—C16—H16B	109.8
H31A—C31—H31B	108.2	H16A—C16—H16B	108.3
C30—C29—C34	109.0 (2)	C11—C13—C12	108.9 (2)
C30—C29—H29A	109.9	C11—C13—C14	109.5 (2)
C34—C29—H29A	109.9	C12—C13—C14	109.3 (2)
C30—C29—H29B	109.9	C11—C13—H13A	109.7
C34—C29—H29B	109.9	C12—C13—H13A	109.7
H29A—C29—H29B	108.3	C14—C13—H13A	109.7
C33—C32—C28	108.9 (2)	C17—C12—C13	109.7 (2)
C33—C32—C31	109.4 (3)	C17—C12—H12A	109.7
C28—C32—C31	109.4 (2)	C13—C12—H12A	109.7
C33—C32—H32A	109.7	C17—C12—H12B	109.7
C28—C32—H32A	109.7	C13—C12—H12B	109.7
C31—C32—H32A	109.7	H12A—C12—H12B	108.2
C32—C33—C34	109.7 (2)	N1—C7—C6	110.9 (2)
C32—C33—H33A	109.7	N1—C7—H7A	109.5
C34—C33—H33A	109.7	C6—C7—H7A	109.5
C32—C33—H33B	109.7	N1—C7—H7B	109.5
C34—C33—H33B	109.7	C6—C7—H7B	109.5
H33A—C33—H33B	108.2	H7A—C7—H7B	108.1
C33—C34—C29	109.8 (2)	C5—C6—C1	117.8 (2)
C33—C34—C27	109.7 (2)	C5—C6—C7	121.7 (2)
C29—C34—C27	109.0 (3)	C1—C6—C7	120.5 (2)
C33—C34—H34A	109.4	O1—C1—C2	118.4 (2)
C29—C34—H34A	109.4	O1—C1—C6	120.8 (2)
C27—C34—H34A	109.4	C2—C1—C6	120.8 (2)
C25—C27—C34	109.6 (2)	C3—C2—C1	120.1 (3)
C25—C27—H27A	109.7	C3—C2—H2C	119.9
C34—C27—H27A	109.7	C1—C2—H2C	119.9
C25—C27—H27B	109.7	C4—C3—C2	120.0 (3)
C34—C27—H27B	109.7	C4—C3—H3A	120.0
H27A—C27—H27B	108.2	C2—C3—H3A	120.0
C25—C28—C32	110.8 (2)	C3—C4—C5	119.7 (3)
C25—C28—H28A	109.5	C3—C4—H4A	120.1
C32—C28—H28A	109.5	C5—C4—H4A	120.1
C25—C28—H28B	109.5	C4—C5—C6	121.6 (3)
C32—C28—H28B	109.5	C4—C5—H5A	119.2
H28A—C28—H28B	108.1	C6—C5—H5A	119.2
			
C23—C22—C21—C20	1.0 (5)	C7—N1—C8—C9	73.8 (3)
C22—C21—C20—C19	−0.7 (5)	C7—N1—C8—C11	−167.8 (2)
C21—C20—C19—C18	0.9 (5)	C7—N1—C8—C10	−48.9 (3)
C20—C19—C18—O2	−179.9 (3)	N1—C8—C11—C13	−179.6 (2)
C20—C19—C18—C23	−1.4 (5)	C9—C8—C11—C13	−59.5 (3)
C21—C22—C23—C18	−1.3 (4)	C10—C8—C11—C13	58.7 (3)
C21—C22—C23—C24	174.0 (3)	N1—C8—C10—C15	−176.5 (2)
O2—C18—C23—C22	−180.0 (2)	C9—C8—C10—C15	59.4 (3)
C19—C18—C23—C22	1.5 (4)	C11—C8—C10—C15	−58.9 (3)
O2—C18—C23—C24	4.6 (4)	C8—C10—C15—C14	60.3 (3)
C19—C18—C23—C24	−173.9 (3)	C8—C10—C15—C16	−60.4 (3)
C25—N2—C24—C23	170.4 (2)	C16—C15—C14—C13	60.0 (3)
C22—C23—C24—N2	139.9 (3)	C10—C15—C14—C13	−60.2 (3)
C18—C23—C24—N2	−44.9 (4)	N1—C8—C9—C17	176.2 (2)
C24—N2—C25—C28	168.6 (2)	C11—C8—C9—C17	59.2 (3)
C24—N2—C25—C27	−73.6 (3)	C10—C8—C9—C17	−58.9 (3)
C24—N2—C25—C26	48.9 (3)	C8—C9—C17—C12	−60.1 (3)
C30—C26—C25—N2	176.7 (2)	C8—C9—C17—C16	59.9 (3)
C30—C26—C25—C28	58.7 (3)	C14—C15—C16—C17	−60.1 (3)
C30—C26—C25—C27	−59.7 (3)	C10—C15—C16—C17	60.2 (3)
C25—C26—C30—C29	60.5 (3)	C12—C17—C16—C15	59.9 (3)
C25—C26—C30—C31	−59.8 (3)	C9—C17—C16—C15	−60.0 (3)
C26—C30—C31—C32	60.1 (3)	C8—C11—C13—C12	59.8 (3)
C29—C30—C31—C32	−59.8 (3)	C8—C11—C13—C14	−59.6 (3)
C26—C30—C29—C34	−60.7 (3)	C15—C14—C13—C11	59.9 (3)
C31—C30—C29—C34	59.2 (3)	C15—C14—C13—C12	−59.3 (3)
C30—C31—C32—C33	59.8 (3)	C16—C17—C12—C13	−60.1 (3)
C30—C31—C32—C28	−59.5 (3)	C9—C17—C12—C13	60.1 (3)
C28—C32—C33—C34	59.8 (3)	C11—C13—C12—C17	−59.9 (3)
C31—C32—C33—C34	−59.8 (3)	C14—C13—C12—C17	59.6 (3)
C32—C33—C34—C29	59.9 (3)	C8—N1—C7—C6	−169.79 (19)
C32—C33—C34—C27	−59.8 (3)	N1—C7—C6—C5	−138.9 (2)
C30—C29—C34—C33	−59.3 (3)	N1—C7—C6—C1	43.7 (3)
C30—C29—C34—C27	61.0 (3)	C5—C6—C1—O1	−179.5 (2)
N2—C25—C27—C34	−175.0 (2)	C7—C6—C1—O1	−2.1 (4)
C28—C25—C27—C34	−58.9 (3)	C5—C6—C1—C2	0.1 (4)
C26—C25—C27—C34	59.6 (3)	C7—C6—C1—C2	177.6 (2)
C33—C34—C27—C25	59.4 (3)	O1—C1—C2—C3	179.7 (2)
C29—C34—C27—C25	−60.9 (3)	C6—C1—C2—C3	0.0 (4)
N2—C25—C28—C32	179.0 (2)	C1—C2—C3—C4	−0.4 (4)
C27—C25—C28—C32	60.0 (3)	C2—C3—C4—C5	0.6 (4)
C26—C25—C28—C32	−58.2 (3)	C3—C4—C5—C6	−0.5 (4)
C33—C32—C28—C25	−60.5 (3)	C1—C6—C5—C4	0.1 (4)
C31—C32—C28—C25	59.1 (3)	C7—C6—C5—C4	−177.4 (2)

### Hydrogen-bond geometry (Å, °)

**Table:**

*D*—H···*A*	*D*—H	H···*A*	*D*···*A*	*D*—H···*A*
O2—H2B···N2	0.82	1.95	2.690 (3)	151
O1—H1B···N1	0.82	1.92	2.670 (3)	152
N2—H2A···O2^i^	0.89	2.64	3.496 (3)	161
N1—H1A···O1^i^	0.89	2.50	3.344 (3)	158

Symmetry codes: (i) *x*, −*y*+3/2, *z*+1/2.
